# The effects of a land-based warm-up and accompanying passive heat retention on core body temperature, hormones, and subsequent performance in elite surfers

**DOI:** 10.3389/fspor.2024.1458268

**Published:** 2024-10-10

**Authors:** Christian J. Cook, Benjamin G. Serpell, Lauren J. Hanna

**Affiliations:** ^1^ School of Science and Technology, University of New England, Armidale, NSW, Australia; ^2^ Hamlyn Centre, Imperial College, London, United Kingdom; ^3^ Geelong Cats Football Club, Geelong, VIC, Australia

**Keywords:** surfing, warm-up, testosterone, cortisol, performance

## Abstract

Surfing is a high participation sport, yet little sport science research exists regarding competitive performance in surfing. Given surfing's inclusion as an Olympic sport from the 2020 Tokyo Olympics onwards, an examination of performance would seem useful. In numerous land-based sports, and in swimming, the importance of a warm-up and muscle heat is well documented. However, surfing is a unique sport in that it is undertaken both above and below water. Therefore, the aim of this study was to explore the effectiveness of a warm-up in terms of readiness to perform in surfing. We discuss this in the context of thermal regulation, hormone profile change, and the subsequent expression of “power” on waves—a key criteria that surfers are scored for. Nineteen advanced level surfers (i.e., competitive at just below national level in Australia; *n* = 15 males and *n* = 4 females) with mean (±SD) age, height, and weight of 24.5 ± 11.6 years, 174.7 ± 9.1 cm, and 67.7 ± 10.2 kg, respectively, were recruited. We adopted a repeated measures pre- and post-design whereby participants engaged in several simulated surfing competitions in an artificial wave pool; once after an active warm-up combined with a passive heat retention strategy (i.e., wrapping themselves in survival blankets—treatment), and once after no warm-up (control). Saliva samples were collected pre- and post-active warm-up, or at equivalent times under control conditions, for the measurement of testosterone and cortisol. Increases in these hormones have previously been associated with an enhanced readiness to compete. Our results demonstrate a clear thermoregulatory benefit from the treatment, with the participants’ core body temperatures typically higher from the end of the warm-up to the end of the surf session following treatment (*p ≤* 0.03), and a magnitude of increase in core body temperature once in the water that is greater following treatment (*p* = 0.01). A small magnitude upward change in testosterone (*p* = 0.01) and cortisol (*p ≤* 0.001) following warm-up was also observed. Finally, warm-up was associated with an improved wave performance compared with the control, with a 20% increase in the performance score typically observed (*p* *≤* 0.01). We argue that the improved thermal profile may have influenced power and, as such, surfing performance was enhanced.

## 1 Introduction

In Australia, and across the world, surfing is a rapidly growing sport. In Australia alone, between 2019 and 2021, 196,000 new participants registered as competitors with formal surfing clubs (1). Furthermore, from 2016 to 2023, there was a surfing population increase of 46% among people aged 18 years or older (2). Currently, in Australia, nearly 730,000 adults engage in surfing, and it is believed these numbers align with worldwide participation rates for which it is thought that more than 50 million people engage in surf sports (2). Despite these high participation rates, and the increased interest in competition in the sport, sports science research in surfing is far from extensive, including as it relates to competitive performance (3). Given the growing interest in the sport, and surfing's inclusion as an Olympic Sport from the 2020 Tokyo Olympics onwards, an examination of performance, or strategies that may enhance performance, would be useful for competitive surfers.

Surfing is unique in comparison with other sports as it is subject to considerably more performance influences than many other sports, including the requirement for physical exertion both above and below water; with paddling through water, surfing on water, disembarking the board, and duck diving under waves (3). In competitive surfing, “power” appears to be critical for performance (4, 5); surfers use aspects of the board (fins and rails) to drive into and out of the water, often producing spray (which anecdotally judges use visually to help assess power). In power-demanding sports, warm muscles, achieved both actively and passively, appear to contribute to an individual’s ability to express power and speed. This has been repeatedly observed in bob skeleton, swimming, rugby union, rugby league, weightlifting, and other sports and across males and females (6–10). As muscle temperature gradually falls, this advantage diminishes. Interestingly, even in very warm climates, muscle warmth, particularly in the legs, and when combined with torso precooling, can provide a marked advantage in power (11). Limited data also suggest that the development of land-based power contributes to surfing prowess in recreational surfers (2, 12). Thus, a warm-up is important for subsequent performance, likely including in surfing, and this notion is supported by a meta-analysis that shows that 79% of research has demonstrated an improvement in performance with a warm-up (13–15). However, for surfing, because data are limited, and owing to its unique nature of requiring physical exertion above and below water, further research is needed to establish how closely knowledge of warm-up from other sports translates to it.

Two further features, in addition to the demonstration of power for judging criteria, of competitive surfing make the warm muscle hypothesis attractive. First, surfing is a limited timed event judged by the total of the best two scored waves (the number of waves allowed varies depending on the competition, from 10 to unlimited). A common and reasonably robust strategy is to try and gain two wave scores early following entry into the water. Thus, an early advantage over opponents of warm muscles, and associated power, would support this strategy. As a contemporary example, Kauli Vaast at the 2024 Paris Olympics, in his gold medal heat, achieved his highest single wave score on his first wave in the heat. Second, competitive surfing is undertaken in water temperatures as low as 7°C and up to 32°C, thus an understanding of muscle and body warmth would be advantageous in warm-up planning. Surfers often complete their main warm-ups somewhat in advance of water entry and anecdotally like to observe water conditions. In other sports in which holding times intervene between the warm-up and the event, passive heat retention has proven to be very useful at maintaining muscle warmth (6) and as such may benefit competitive surfing. In an earlier study, we demonstrated a considerable thermal gain in recreational surfers entering relatively cold water through the combination of an active warm-up and passive heat maintenance (2). This also had some positive outcomes for the way they surfed (e.g., more maneuvers per wave and better pop-ups) (2).

An important component to warm-ups is not just increasing temperature and the potential related muscle power, but also enhancing overall readiness; with readiness being defined as a complete state of physical, behavioral, and psychological preparedness to compete (16). In research, readiness has been linked to the stress hormones cortisol and testosterone, which are possibly biomarkers; however some direct effects may also occur. Cortisol has been reported to be arousing and has been linked to performance in short-duration sporting events, such as in judo in males (17), Olympic weightlifting in males and females (18), and rowing performance (in some contexts) in females (19). However, elevations in cortisol can also lead to aggressive behaviors and increased fatigue and anxiety (16, 17, 20). Testosterone has been linked to enhanced motivation, motor control, the neuromuscular expression of speed and power, assertiveness, and collaborative competitiveness (16, 21–23). Testosterone increases have been linked to performance in longer-duration events and team sports in both males and females including rugby union (24, 25), rugby sevens (26), and hockey (27). The linkage of testosterone, in particular, to speed and power may be of value to power elements in surfing (28). Testosterone and cortisol may therefore be related to the readiness to compete and are often considered relative to each other using the testosterone-to-cortisol ratio (16, 20, 21, 29, 30), and this ratio may have some flow-on effect on recovery. Therefore, warm-up protocols associated with elevations in testosterone may potentially contribute to performance.

The aim of this study was to explore whether a structured warm-up combined with passive heat retention influenced surfing performance. Under simulated competition conditions, and in elite level competitive surfers, performance was measured by judged criteria. We hypothesized that the warm-up and heat retention strategy would elevate core body temperature, and hence muscle temperature, enabling surfers to express more power early in the session and garnish higher judged scores. In addition, we collected saliva before and after an active warm-up and subsequently assayed for testosterone and cortisol to observe whether there were any potential links of hormones to warm-up protocols.

## 2 Materials and methods

### 2.1 Study overview

**Figure 1:**
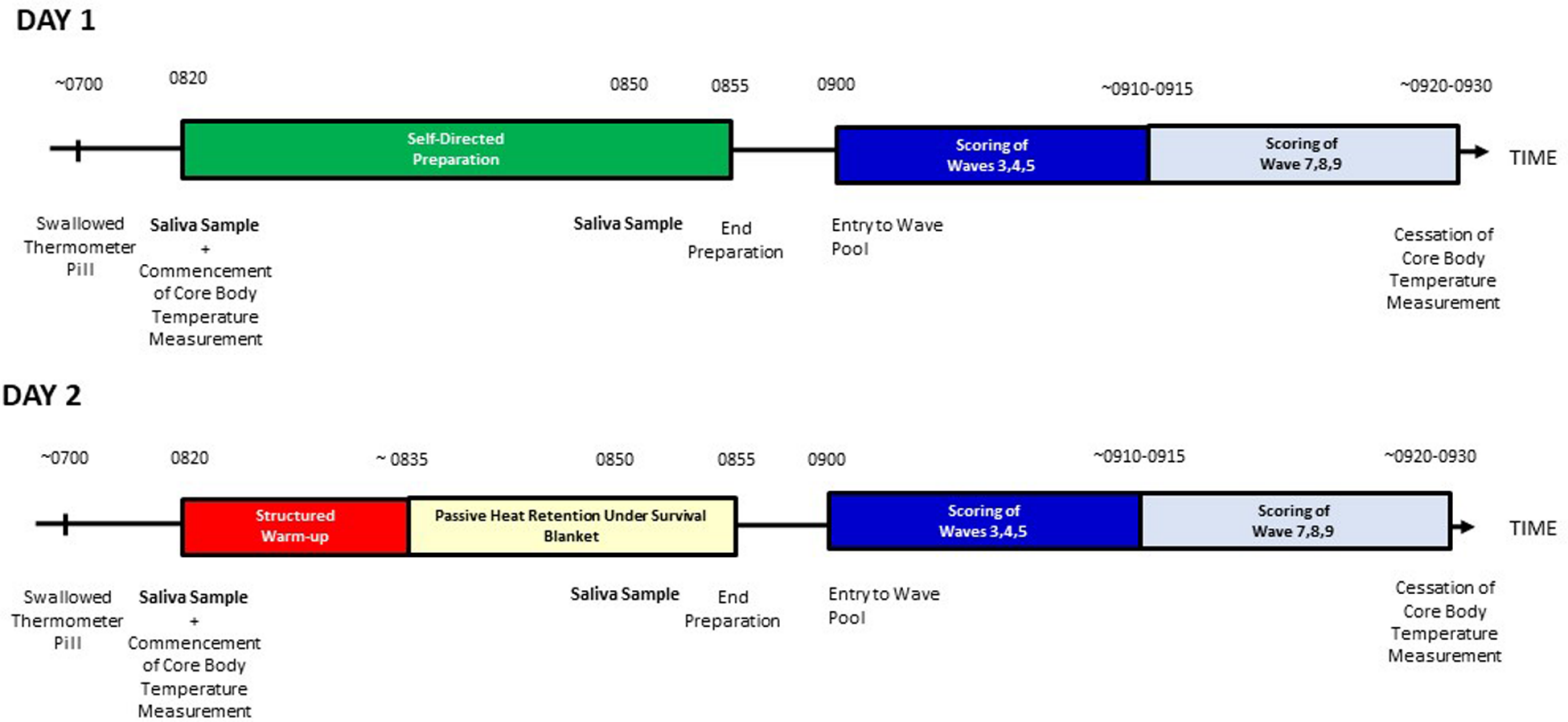
Schematic of the research protocol. Note: sessions were completed 24 h apart.

A schematic of the research protocol can be seen in Figure 1. This study adopted a repeated measures pre- and post-design. Participants attended an artificial wave pool (UBRN Surf, Victoria, Australia) on three occasions—each session was 24 h apart: one attendance was for familiarization, one was for experimental conditions, and one was for “control” conditions. For the “experimental” surf, participants entered the wave pool following a combined active and passive warm-up; for the control, they entered the pool without completing a warm-up. Thus, participants acted as their own control. The benefits behind this approach is that the recruitment of participants is easier, and it reduces the effect of confounding factors. For both control and experimental conditions, participants swallowed a thermometer pill (BMedical, Paris, France) and core body temperature was measured every 30 s for the duration of the data collection. A saliva sample was also collected from each participant immediately before and 15 min after the active warm-up (or an equivalent amount of time under control conditions) for the measurement of testosterone and cortisol. Finally, performance in the subsequent surf sessions (control and experimental conditions) was scored by two Australian state-level qualified surfing judges, blinded to treatment conditions. Hormones, core body temperature, and performance were compared between sessions, and the relationships between them were explored.

### 2.2 Participants

Nineteen participants (*n* = 15 males and *n* = 4 females) were randomly recruited to this study via surfing clubs and community groups in Victoria, Australia, and word of mouth. The means for (±SD) age, height, and weight for the entire group were 24.5 ± 11.6 years, 174.7 ± 9.1 cm, and 67.7 ± 10.2 kg, respectively, and specifically, the male participants were 22.3 ± 10.3 years, 177.6 ± 7.8 cm, and 69.7 ± 10.6 kg, respectively, and the females were 32.8 ± 14.2 years, 164.5 ± 5.3 cm, and 60.0 ± 0.3 kg, respectively. All participants were advanced level surfers (i.e., they were capable of executing aerial maneuvers and were competitive at just below national level in Australia). Ten of the surfers were goofy footed (i.e., they stood on a surfboard with their right foot forward; *n* = 9 males and *n* = 1 female) and nine surfed with a natural stance (i.e., they stood on a surfboard with their left foot forward; *n* = 6 males and *n* = 3 females).

### 2.3 Procedures

Approval to conduct this research was granted by the University of New England Human Research Ethics Committee (protocol number HE22-141). Before the commencement of data collection, voluntary informed consent to participate was provided by each participant following a briefing of the research aims and protocols.

For familiarization, participants surfed at the artificial wave pool in their own time before the commencement of the experiment. Thereafter, on two separate occasions, 24 h apart, participants attended the wave pool at approximately 7:00 a.m. and swallowed a thermometer pill. On the control day, participants were instructed to prepare for a surf as they normally would and entered the pool at 9:00 a.m.. On the experimental day, at 8:20 a.m., participants commenced a land-based warm-up in their wetsuits before covering themselves with a survival blanket until they entered the wave pool at 9:00 a.m. (of note, participants all wore a minimum 3-2 combination wetsuit, i.e., they wore a wetsuit with chest and back panels made of 3 mm neoprene foam, with limb panels made of a 2 mm neoprene foam; they were instructed to wear the same wetsuit on both occasions). The survival blankets were low-weight low bulk blankets made of thin plastic heat-reflective sheeting, which assists with heat attainment and retention.

The warm-up was similar to that described previously (16) and consisted of

1. 2–5 min of general mobility,
2. 2–3 min of upper body and lower body reactive strength exercises,
3. 2–3 min of upper body and lower body elastic strength exercises, and
4. 2–3 min of upper body and lower body mechanical power exercises.

The warm-up took 12–15 min, therefore participants remained under survival blankets, remaining reasonably still for 15–20 min. Saliva samples were collected for hormone measurement from participants at approximately 8:20 and 8:50 a.m. on both days (i.e., just before the commencement of the active warm-up and approximately 15 min after the active component of the warm-up, on the experimental day; and the same times on the control day). Core body temperature was analyzed from 8:20 to 9:40 a.m. on both the experimental and control days (i.e., from the commencement of the warm-up to the end of the surfing session), and data were compared within and between days from the full minute at the commencement of the active warm-up (8:20 a.m.), completion of the active warm-up (approximately 8:35 a.m.), completion of the passive warm-up (8:55 a.m.), when participants entered the pool (9:00 a.m.), at the start of the first competition block (approximately 9:07 a.m.), at the start of the second competition block (approximately 9:20 a.m.), and at the completion of the surf (9:40 a.m.). The magnitude of change between the data points was also compared between days. The water temperature in the wave pool was a consistent 13–16°C each session.

### 2.4 Core temperature measurement

Thermometer pills were activated prior to ingestions using an eCelsius Performance Activator (BMedical, Paris, France) and swallowed by participants 90–60 min before commencing the warm-up. Participants were instructed not to consume drinks between swallowing the pill and entering the pool so that the core temperature readings were not affected while the pill was in their stomach. All temperature readings were transferred wirelessly from participants soon after the completion of the surf using an eCelsius Performance Monitor (BMedical, Paris, France) and downloaded to ePerformance Manager (BMedical, Paris, France) and exported to Microsoft Excel for later analysis.

### 2.5 Hormone measurement

Testosterone and cortisol were measured from saliva samples collected from participants immediately before the commencement of the active warm-up and 15 min after the completion of the active warm-up. It is well known that a lag time exists for hormone change from plasma to saliva (31, 32), with the time being individual and context dependent, but allowing for a lag time of approximately 10–15 min is generally acceptable, hence the second saliva sample was collected 15 min after the completion of the active warm-up. Saliva samples were collected from participants in sterilized cryovials and stored in a −20°C freezer until analysis to ensure the testosterone molecules crystalized and did not break down. They were thawed and assayed for cortisol and testosterone using commercial enzyme immunoassay kits (cortisol, catalog number 1-3002; testosterone, catalog number 1-2402; Salimetrics LLC, State College, PA, USA). Intra- and inter-assay variability was <5.0% for testosterone and cortisol. Samples were analyzed in the same assay to eliminate inter-assay variance.

### 2.6 “Competition” structure and scoring of performance

An advantage of using an artificial wave pool is the ability to control the “type” of wave participants surf and when they surf it. As such, the waves each participant surfed on each occasion were standardized as “advanced” level waves, with the wave face height always 1.6–2.0 m, the likely peel angle at approximately 30° (making it a fast wave), and the wave length at 12–16 s. The wave pool had both left and right breaking waves, and participants surfed on their preferred side for the entire session in every session. Fortuitously, nine participants surfed on the right breaking wave and 10 participants surfed on the left breaking wave. All participants were in the wave pool at the same time and took turns riding waves (i.e., only one person per wave; however, they were all presented with the same waves) (33, 34). All sessions were recorded on video, and performance was scored at a later date by two experienced Australian state-level qualified surfing judges for “flow,” “speed,” “style,” and “power” across the entire wave from “pop-up” to exit according to standardized Surfing Australia protocols (35). Participants were scored in two “blocks” of three waves (waves 3–5 and waves 7–9). The best two scores for each scoring block were also added for an overall score. Thus, three scores were subsequently given—performance in the first scoring block, performance in the second scoring block, and overall performance (which was the best two scores across both scoring blocks). Where scoring differed in this study compared with Surfing Australia protocols is that scores were given on a scale of 0–10 with increments of 0.5, as opposed to scores being continuous in nature between 0 and 10; making the data somewhat categorical in nature. Scoring was given in 0.5 increments because realistically it is difficult to argue a score of smaller increments (e.g., can you really ascertain performance difference when someone is scored 5.5 vs. 5.6?). Scores between judges were averaged, and when the average sat between increments of 0.5, the score was rounded up (e.g., a score of 5.75 was rounded to 6.0). All scored waves were completed within 25 min of getting into the wave pool.

### 2.7 Statistical analysis

#### 2.7.1 Core body temperature

Data were first assessed to establish whether they met the assumptions of parametric statistical analysis (i.e., the Shapiro–Wilk test for normality and Levene's test of equality). These assumptions were not met by all data; as such, a Friedman's test was performed on all data combined, on male only data, and on female only data to establish whether a difference existed between sessions and within sessions for core body temperature. Where significant differences were identified, Wilcoxon signed-rank tests were applied to establish where significant differences existed within and between sessions. We also applied Wilcoxon sign-ranked tests to the magnitude of differences.

#### 2.7.2 Hormones

Similarly to core body temperature, data were first assessed to establish whether they met the assumptions required for parametric statistical analysis. Again, the assumption were not met; therefore, a Friedman's test was performed on all the data combined, on male only data, and on female only data to establish whether a difference existed between sessions and within sessions for testosterone, cortisol, and the testosterone-to-cortisol ratio. Where significant differences were identified, Wilcoxon signed ranked tests were applied to establish where significant differences existed within and between sessions.

#### 2.7.3 Performance

Given the categorical nature of the data, a non-parametric statistical analysis was most suitable. As such, a Friedman's test and Wilcoxon signed-rank tests were also applied to the performance data. All statistical analyses were performed using Statistical Package for Social Sciences software version 29.0 (IBM, New York, NY, USA) (*α* = 0.05).

## 3 Results

Median and interquartile ranges can be seen for the core body temperature data in Table 1. Friedman's test on data for all participants for core body temperature revealed a significant difference [*χ*^2^(13) = 141.11, *p <* 0.001]. A significant difference was also observed for the male only [*χ*^2^(13) = 103.39, *p <* 0.001] and female only data [*χ*^2^(13) = 33.47, *p =* 0.001].

**Table 1:** The core body temperatures in degrees Celsius of participants during the warm-up (or control) and surfing session.

	Start of the warm-up	End of the active warm-up	End of the passive warm-up	Pool entry	Start of the first competition set of waves	Start of the second competition set of waves	End of the surf session
All participants							
Warm-up	37.38 ± 0.53	37.45 ± 0.38^a^^,^^b^	37.33 ± 0.36	37.39 ± 0.41	37.78 ± 0.41^a^^,^^c^	38.13 ± 0.55^a^^,^^c^	38.25 ± 0.32^d^
Control	37.10 ± 0.42	37.26 ± 0.48^b^	37.25 ± 0.43	37.25 ± 0.43	37.45 ± 0.51^c^	37.84 ± 0.49^c^	38.02 ± 0.71^e^
Males							
Warm-up	37.24 ± 0.56	37.38 ± 0.42^a^^,^^b^	37.33 ± 0.38	37.32 ± 0.41	37.72 ± 0.35^a^^,^^c^	38.00 ± 0.41^a^^,^^c^	38.15 ± 0.37^d^
Control	37.02 ± 0.40	37.25 ± 0.42^b^	37.18 ± 0.36	37.17 ± 0.24	37.38 ± 0.37^c^	37.76 ± 0.54^c^	38.02 ± 0.68^e^
Females							
Warm-up	37.45 ± 0.17	37.52 ± 0.18	37.41 ± 0.24	37.45 ± 0.34	37.86 ± 0.27	38.33 ± 0.14	38.30 ± 0.14
Control	37.30 ± 0.27	37.49 ± 0.12	37.45 ± 0.53	37.44 ± 0.34	37.79 ± 0.10	37.99 ± 0.26	37.95 ± 0.48

Data are median ± interquartile range.

^a^ Significantly greater than the same time point compared with the control (*p* < 0.05).

^b^ Significantly greater than the start of the warm-up (*p* < 0.05).

^c^ Significantly different to all time points prior (*p* < 0.05).

^d^ Significantly greater than all the prior time points but not the start of second set of competition waves (*p* < 0.05).

^e^ Significantly greater than all the time points prior, including the second competition set of waves (*p* < 0.05).

Wilcoxon signed-rank tests revealed that for the entire group data, core body temperature under warm-up conditions was greater than that under control conditions at the end of the active warm-up (*Z* = 2.22, *p =* 0.03) at the start of the first competition set of waves (*Z =* 2.74, *p =* 0.01) and at the start of the second competition set of waves (*Z =* 2.95, *p <* 0.01). When exploring male only data, the difference was only observed at the start of the first competition set (*Z =* 2.59, *p =* 0.01) and at the start of the second competition set (*Z* = 2.28, *p =* 0.02). For female only data, no significant differences between conditions were observed. When looking at experimental group differences within sessions, the core body temperature increased following the active warm-up (*Z* = 2.48, *p* = 0.01). Core body temperature was also greater at the start of the first and second competition blocks and at the end of the surfing session than at the end of the active and passive warm-ups and at pool entry (*Z >* 3.46, *p* *<* 0.01). It also increased from the start of the first competition set to the second competition set, and from the start of the first competition set to the end of the session (*Z* ≥ 3.15, *p* ≤ 0.01). Similar observations were made under control conditions (*Z =* 2.72*, p* = 0.01) when comparing competition sets and the end of the surfing session to the start of the surfing session, to the end of the active warm-up component, end of the passive warm-up component, and pool entry (*Z* ≥ 2.64, *p <* 0.01). Core body temperature was also greater at the start of the second competition block and at the end of the surfing session compared to the first competition block (*Z >* 2.64, *p* ≤ 0.01). Interestingly, core body temperature was greater at the end of the session than at the start of the second competition wave set under control conditions (*Z =* 2.12, *p* = 0.03), but not under experimental conditions. Broadly speaking, similar observations were made for the male only data (*Z* ≥ 2.24, *p* ≤ 0.05). Comparisons within sessions did not reach statistical significance for the women only data (Table 1).

**Figure 2:**
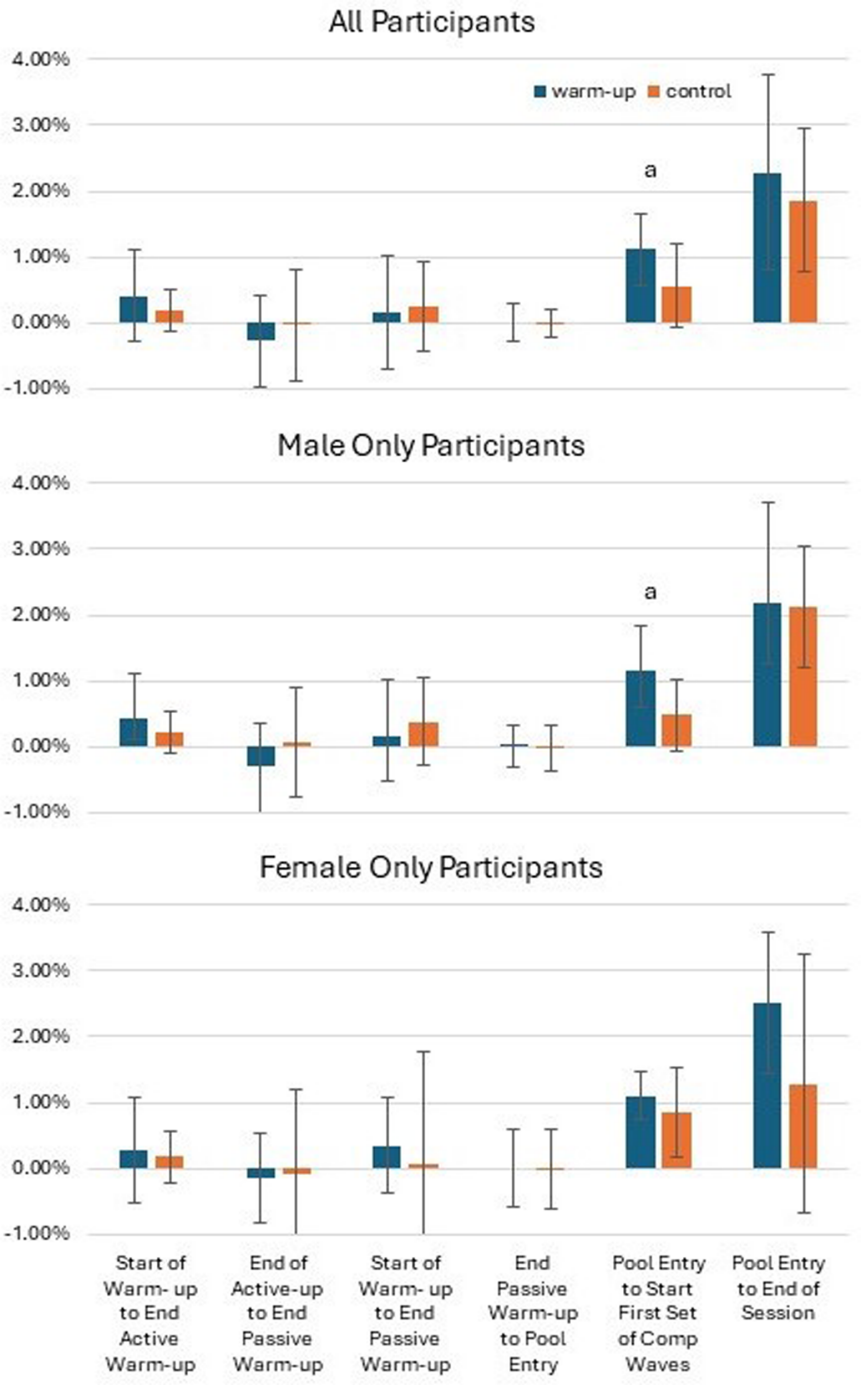
Change in the core body temperature as a proportion of the baseline (start of the warm-up) core body temperature. Data are median ± interquartile range. ^a^Magnitude of increase significantly greater for warm-up (*p* < 0.05).

When exploring differences in the magnitude of increase in core body temperature, the data from all participants combined showed that the magnitude of increase in core body temperature from pool entry to the first competition set was greater following warm-up than in the control (*Z* = 2.84, *p* ≤ 0.01). This was similarly observed in the male only data (*Z =* 2.43, *p* = 0.02), but the female only data did not reach statistical significance (Figure 2).

**Figure 3:**
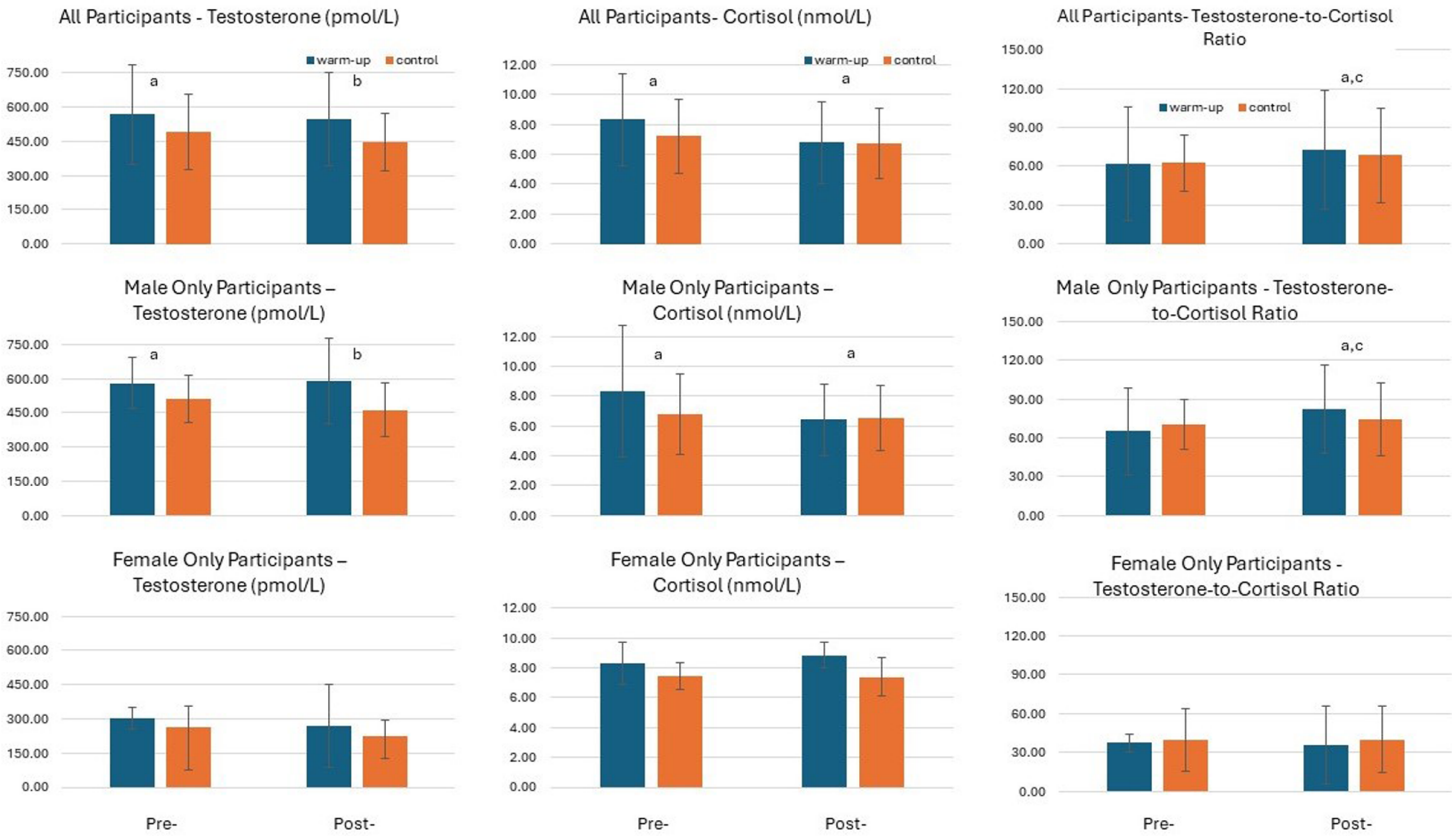
Cortisol, testosterone, and the testosterone-to-cortisol ratio before and 15 min after the active warm-up or at equivalent times under control conditions (i.e., 8:20 and 8:50 a.m., respectively). Data are median ± interquartile range. ^a^Significantly greater under warm-up conditions than under control conditions (*p* < 0.05). ^b^Significantly greater post-warm-up than pre-warm-up (*p* < 0.05). ^c^Significantly greater post than pre under control conditions (*p* < 0.05).

For hormone data, the Friedman's test on all participant data revealed a significant difference for testosterone [*χ*^2^(3) = 11.19, *p =* 0.01] and cortisol [*χ*^2^(3) = 18.35, *p* ≤ 0.001], but not for the testosterone-to-cortisol ratio. Similar observations were made for the male only data [*χ*^2^(3) = 8.15, *p =* 0.04 and *χ*^2^(13) = 14.12, *p* ≤ 0.01, respectively], but, again, no statistical significance was reached for the female only data. Wilcoxon signed-rank tests revealed that, for all participants, testosterone was greater at the start of the day before the warm-up than at the start of the day under control conditions (*Z* = 2.25, *p* = 0.02), and at post-active warm-up compared with pre-active warm-up (*Z =* 2.54, *p* = 0.01). Cortisol was also greater under warm-up conditions than under control conditions pre-warm-up (*Z* = 2.90, *p* ≤ 0.01) and post-warm-up (*Z* = 3.10, *p* ≤ 0.01). Finally, the testosterone-to-cortisol ratio was greater post-warm-up than pre-warm-up (*Z* = 1.93, *p* = 0.05), and post-warm-up compared to pre-warm-up under control conditions (*Z* = 1.81, *p* = 0.05). Similar observations were made for the male only data (*Z* = 1.99, *p* = 0.05; *Z* = 2.39, *p* = 0.02; *Z* = 2.56, *p* = 0.01; *Z* = 2.61, *p* ≤ 0.01; *Z* = 2.25, *p* = 0.03; and *Z* = 1.99, *p* = 0.05, respectively) (see Figure 3).

**Figure 4:**
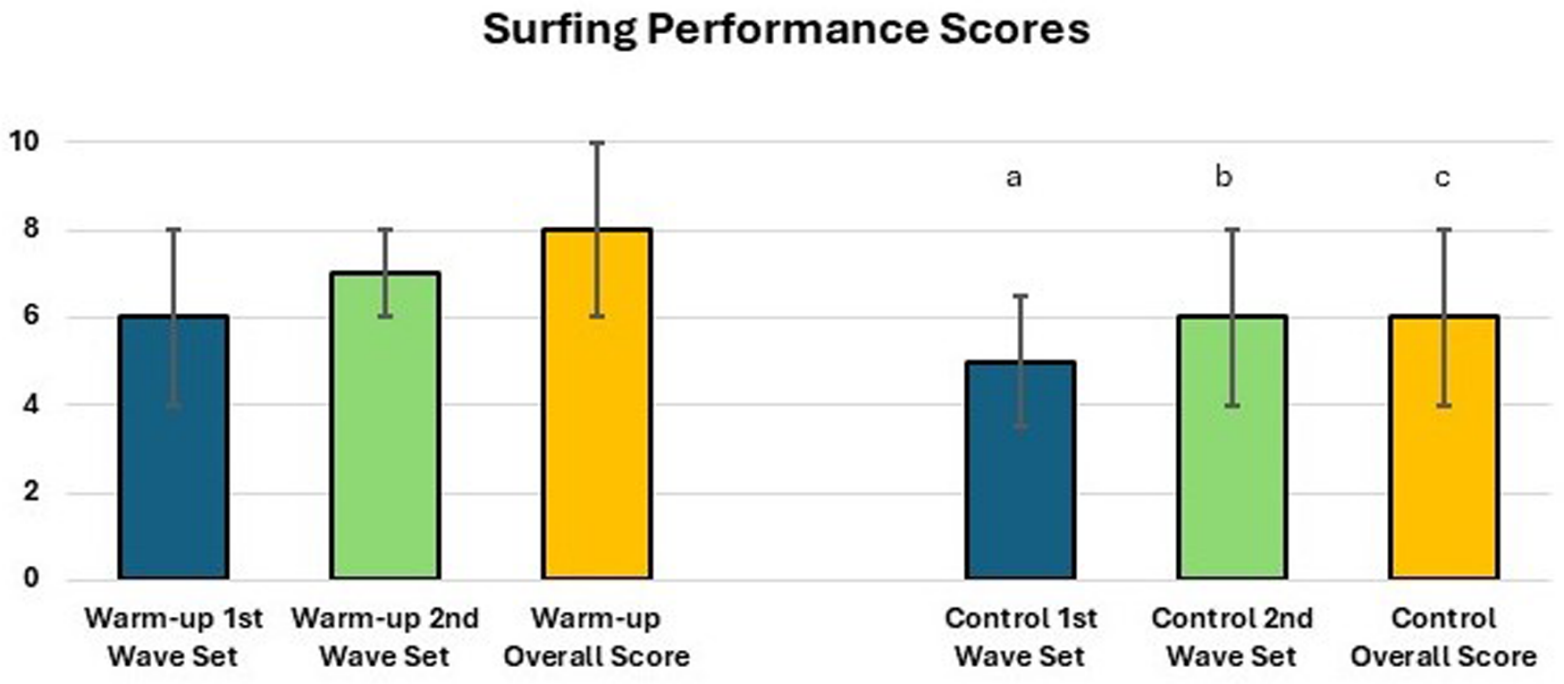
Median scores given to participants for the first set of waves and second set of waves and an overall score (i.e., the score for both sets of waves combined) under warm-up conditions and control conditions. ^a^Significantly lower than the first wave set under warm-up conditions overall and for male participants (*p* < 0.05). ^b^Significantly lower than the second wave set under warm-up conditions overall and for male participants (*p* < 0.05). ^c^Significantly lower than the overall score under warm-up conditions overall and for male participants (*p* < 0.05).

Finally, Friedman's test for the performance data for all participants combined also revealed a significant difference for the first competition set, second competition set, and overall score for warm-up compared with control conditions [*χ*^2^(5) = 49.70, *p* ≤ 0.001]. This was similarly the case for the male only data [*χ*^2^(5) = 38.51, *p* ≤ 0.01], but statistical significance was not reached in the female only data. Wilcoxon signed-rank tests showed the performances for the first wave set and second wave set, and the overall score for the first wave set, were better than for the equivalent in the second wave set (*Z* > 2.41, *p* ≤ 0.02). Similar observations were made for the male only data (*Z* > 1.95, *p* ≤ 0.05) (see Figure 4). Aside from the significant findings noted above, and demonstrated in the tables and figures, no other differences were observed between conditions.

## 4 Discussion

The purpose of this study was to explore whether surfing performance is influenced by a structured warm-up combined with passive heat retention. We anticipated that the warm-up and heat retention strategy would elevate core body and hence muscle temperature, enabling surfers to express more power earlier and achieve higher judged scores. Our results demonstrated a clear thermal advantage to an active warm-up and passive heat retention. Interestingly, this advantage lasted for more than 25 min of surfing in relatively cool water. This was similar to the thermal profiles we reported for recreational surfers at comparable water temperatures (2).

In the present study, the magnitude of core body temperature increase was greater and “more rapid” following warm-up than under control conditions after participants entered the water, and were active paddling, but reached a plateau earlier. This was further highlighted by the fact that under control conditions there remained a significant difference in core temperature from the start of the second competition block to the end of the session, but not under warm-up conditions, and by the end of the pool session, the core temperature for both conditions was similar. Our study also demonstrated that, secondary to the improved thermal advantage, there was a small change in hormonal profile following active warm-up, with an increase in testosterone being observed and a clear decline in cortisol being demonstrated following active warm-up, along with significant differences within subjects between conditions. Finally, performance, as judged by surfing Australia criteria using two independent judges blinded to treatment, showed a consistent difference across both competition sets of wave blocks and subsequent overall best two wave score with warm-up and passive heat retention being associated with higher scores.

Our results were not unexpected given the thermal effect of active warm-up and the passive heat retention properties mirroring results presented in other sports (6–10, 36), and our active warm-up protocol also achieved similar results in terms of hormonal change (2, 16). In fact, other sports studies, in which an appropriate warm-up has been explored, have produced quite compelling evidence that has been instrumental in forwarding an argument for warm-ups and understanding the effect of thermal profiles on surfing performance (36). Surfing competitions can also take place in warm water, and on land, when ambient conditions are hot and humid, precooling can influence performance positively. Recent research has shown that in repeat sprint land models in very hot environments where precooling can have a positive effect on performance, combining the concepts of warm legs (through active and passive warming) and a cool body (precooling) may offer a competitive advantage greater than either alone, suggesting this is worthy of exploration in surfing performance (11).

A novel finding from this present study was that the gain in thermal properties mirrored performance improvement across waves, and the advantage lasted at least 25 min in the water; given the small number of participants, this was a strong finding. In general, in land-based sports, the thermal advantages of warm-up dissipate 10–15 min into competition (36). However, surfers are both above and in the water, and in this case were wearing a minimum of 3–2 mm combination wetsuits. Water exposure and the wetsuit offer slightly different properties to those seen on land. Such a period of thermal gain therefore has strong implications for the entire period of a competitive heat duration in surfing, which would typically be between 15 and 30 min.

As noted earlier, there were some small differences in hormone levels between the warm-up and control treatment with both testosterone and cortisol trending higher in the warm-up protocol. There are several possible reasons for the increase in hormone magnitude. First, athletes may have been more anticipatory aroused prior to knowing they were doing a warm-up protocol, and the warm-up itself is short and intense, which can activate hormones (7). Potentially, one benefit of this may be the increased availability of power and speed (28, 37, 38), which could conceivably have also influenced surf power performance. Changes in hormones may also facilitate mental states of assertiveness, self-belief, and competitiveness in males and females (39, 40). Finally, it is worth noting that the testosterone-to-cortisol ratio at the conclusion of competition in other sports has been linked to a more rapid and “better” recovery (41). The magnitude of change we observed in this study was possibly too small to effectuate these changes; however, further investigation is merited.

Despite our research demonstrating the value of warm-ups for surfing performance, it is worth noting that in work in bob skeleton, athletes adopted warm-ups and passive heat strategies that had a thermal advantage, which also best suited them (6). As such, in that research, the adoption of practice was sometimes not necessarily optimal in terms of increasing the measured sprint-power performance, but it did provide participants with the best sense of overall performance. In fact, all took some advantage speed and power from their own adapted warm-up. This highlights the point that most sports have other elements aside power and speed that must be balanced into a successful performance equation, and why it remains valuable to monitor the hormonal response to warm-up (as they may indicate the mental state). For example, athletes are often anecdotally fashion conscious (42–44) and choose their performance clothing with this in mind; therefore, good clothing design incorporating elements of thermal gain will likely be an important contributor longer term to any strong broad compliance.

Thermal comfort and individual perception is an interesting factor that may influence clothing choice in surfing. For example, neoprene wetsuits can influence thermoregulation (45) and have been shown to have buoyancy properties that may enhance performance (46); however, research has shown that wetsuits more than 2 mm thick can affect arm motion in surfers (47), and, speculatively, performance. Furthermore, it has been shown elsewhere that synthetic garments that enhance comfort can sometimes trump performance-based choices in terms of the adoption of a practice (43, 44, 48). Thus, it becomes clear that factors other than performance gains can influence the likelihood of the uptake of thermoregulation strategies (e.g., comfort and esthetics). Therefore, the findings from this study may prove useful when it comes to designing clothing that enhances thermoregulation and, by extension, surfing performance by using different materials on different regions of the wetsuit so as not to restrict movement, while also considering that some neoprene is useful for maintaining the core body temperature and muscular warmth. Wetsuit manufacturers already do this to a degree by using mixed composite materials to try and combine stretch, comfort, and warmth; these data could further enhance that thinking. There may also be challenges associated with maintaining skin temperature in regions of the body most likely to make contact with the water (e.g., calves and thighs) (45), highlighting a further need for the integration of different materials across wetsuits to maintain the core and muscular temperature. Combined, wetsuits with such novel designs, for example, may ensure the maintenance of the core body temperature and provide a performance advantage provided they are esthetically pleasing.

There are several caveats in this study, fatigue being one. Although the athletes did not report any fatigue across the different sessions, this remains a confounder that we did not adequately dissect out in our study due to practical limitations, such as athlete availability and cost. In addition, hormone differences between treatments were small and preliminary in nature only, and underpowered in female number. They simply suggest an avenue for more exploration. We believe that both gender and the BMI may be important, particularly in clothing design. We did not have the numbers or spread in our study to elucidate if this is so. In particular, our female numbers were underpowered; more female studies are clearly needed. The ability to measure actual power or speed would further the science of this study, although ultimately, in sport, the impact on measured performance (in this case, wave scores) is primary. As mentioned, athlete compliance, adoptability, and practicality are essential features in a true competitive ecological environment. Our data provide some foundations to further this and suggest that an understanding of active warm-up, passive heat retention, and any associated physiological changes such as hormone status may have considerable performance benefit in surfing.

## 5 Conclusion

In a simulated competition context, this study showed a clear thermoregulation benefit of adopting a land-based warm-up with a passive heat retention strategy (i.e., being wrapped in a survival blanket) prior to surfing. Unsurprisingly, therefore, we saw a better performance in surfers following the land-based warm-up with passive heat retention, likely due to the muscle power availability that has been associated with body warmth. Surprisingly, the benefit lasted longer than observed in land-based studies and was noted in every participating athlete. Secondary to this, we saw small magnitude differences in the hormones testosterone and cortisol that speculatively could relate to a readiness to compete. These findings have clear implications for competitive surfing, and also, potentially, for product development (e.g., wetsuits and other warming garments).

## Data Availability

The datasets presented in this article are not readily available because of Ethics Committee requirements. Requests to access the datasets should be directed to ben.serpell@gmail.com.
